# Immune dysregulation and system pathology in COVID-19

**DOI:** 10.1080/21505594.2021.1898790

**Published:** 2021-03-23

**Authors:** Muhammad Jamal, Hina Iqbal Bangash, Maria Habiba, Yufei Lei, Tian Xie, Jiaxing Sun, Zimeng Wei, Zixi Hong, Liang Shao, Qiuping Zhang

**Affiliations:** a Department of Immunology, School of Basic Medical Science, Wuhan University, Wuhan P.R. China; b State Key Laboratory of Agricultural Microbiology, College of Life Science and Technology, Huazhong Agricultural University, Wuhan China; c Department of Zoology, University of Malakand, Chakdara Dir Lower, Khyber Pakhtunkhwa Pakistan; d Department of Hematology, Zhongnan Hospital of Wuhan University, Wuhan P.R. China; e Hubei Provincial Key Laboratory of Developmentally Originated Disease, Wuhan University, Wuhan P.R. China

**Keywords:** SARS-COV-2, covid-19, cytokine release syndrome, lymphopenia, pathogenesis, ards and multiple organs failure

## Abstract

The coronavirus disease 19 (COVID-19) caused by the novel coronavirus known as SARS-CoV-2 has caused a global public health crisis. As of 7 January 2021, 87,640,402 confirmed cases and 1,891,692 mortalities have been reported worldwide. Studies focusing on the epidemiological and clinical characteristics of COVID-19 patients have suggested a dysregulated immune response characterized by lymphopenia and cytokine storm in these patients. The exaggerated immune response induced by the cytokine storm causes septic shock, acute respiratory distress syndrome (ARDS), and/or multiple organs failure, which increases the fatality rate of patients with SARS-CoV-2 infection. Herein, we review the recent research progress on epidemiology, clinical features, and system pathology in COVID-19. Moreover, we summarized the recent therapeutic strategies, which are either approved, under clinical trial, and/or under investigation by the local or global health authorities. We assume that treatments should focus on the use of antiviral drugs in combination with immunomodulators as well as treatment of the underlying comorbidities.

## Background of COVID-19

**Figure 1.**
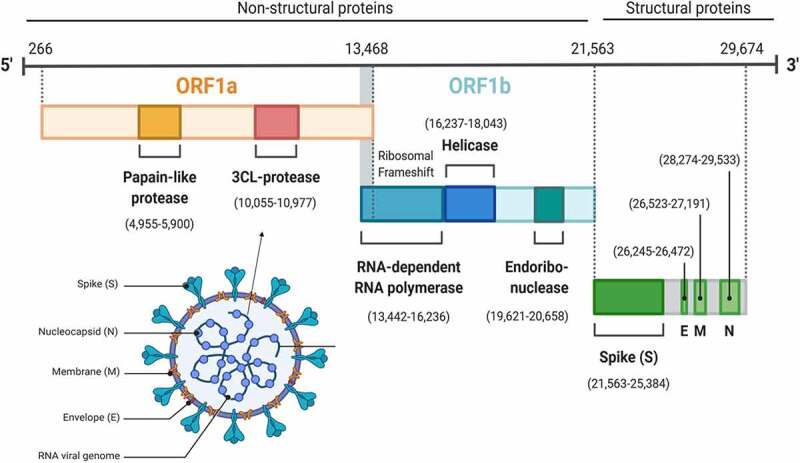
**Structure and genome organization of SARS-CoV-2 particle**. (a) SARS-CoV-2 is a large pleomorphic, nonsegmented, enveloped, and spherical round virus with projections. The structure is composed up of a lipid bilayer in which M, E and S proteins are embedded. The lipid bilayer encloses nucleocapsid, which is made up of multiple copies of N protein binding wrapping around genomic RNA. (b) The genomic RNA is positive-sense and single-stranded, which is 29kb. Two-third of the genome contains open reading frames (ORF1a and ORF1b), which encode the non-structural proteins essential for viral replication and viral protein synthesis. The 5ʹ and 3ʹ ends of the genome contain a methylated cap and poly-A tail, respectively

On 30 January 2020, the world health organization (WHO) declared the coronavirus disease 19 (COVID-19) as a public health emergency of international concern (PHEIC), and declared it as a pandemic on 11 March 2020 [1–3]. Globally, 210 countries are being affected by the SARS-CoV-2 (Figure 1). The total number of incidence and mortalities, global distribution of COVID-19 cases as of 29 May are summarized in Figure 1. The virus can be transmitted among persons via aerosol or fomites [4]. The most common symptoms of COVID-19 are fever, cough, expectoration, fatigue, dyspnea, conjunctivitis, myalgia, as well as pneumonia. The less observed clinical symptoms are headache, hemoptysis, and diarrhea [5]. The majority of cases (up to 80%) develop mild symptoms or remain asymptomatic, while up to 10–20% of the patients develop severe pneumonia. Approximately 5% of the cases develop ARDS, septic shock, and multiple organs failure [5–8].

The mechanism of COVID-19 is not fully understood yet. Based on recently published immunological data, COVID-19 patients have a dysregulated immune response, characterized by the release of multiple inflammatory cytokines, such as tumor necrosis factor (TNF), interleukins, as well as a decreased number of T cells, B cells, and natural killer (NK) cells [8]. In order to design effective therapeutic interventions for COVID-19, a better understanding of its underlying mechanism about immune dysregulation is of paramount importance. In this review, we will focus on the general characteristics of SARS-CoV-2, immune dysregulation, and pathophysiology of COVID-19. In addition, a list of potential drugs will be demonstrated, which are either under investigation, in the clinical trial, or being approved by the health authorities.

## Characteristics of Coronavirus

Coronaviruses were initially discovered in the mid of 1960s and have caused fatal respiratory illness over the last two decades, ranging from common cold to Middle East Respiratory Syndrome (MERS) and Severe Acute Respiratory Syndrome (SARS) [9]. SARS-CoV-2 is a member of the β-coronavirus cluster from subfamily orthocoronavirinae, family coronviridae, and subgenus sarbecovirus, the most prevalent classes of the virus in nature. SARS-CoV-2 is an enveloped virus with a positive-sense single-stranded enveloped RNA virus and a helically symmetrical nucleocapsid [10].

### Genome size and structure

The size of SARS-CoV-2 genome is 29.9 kb [11], which is within the range of the size of other coronavirus (26–32 kb) [12]. The 5ʹ and 3ʹ prime end of the genome has a methylated cap and a polyadenylated tail, respectively [13]. Likewise, in other betacoronaviruses, the genome structure of SARS-CoV-2 is composed of open reading frames (ORF1a and ORF1b), which contain non-structural genes essential for replication and viral protein synthesis. Structural proteins such as spike surface glycoprotein (S), small envelope protein (E), matrix protein (M), nucleocapsid protein (N) are located near the 3ʹ-UTR [13–15]. Genome sequencing analysis of the SARS-CoV-2 revealed 79% and 96% similarity with SAR-CoV-1 and bat SARS coronavirus, respectively [16]. Interestingly, the genomic sequencing analysis of SARS-CoV-2 from Japan, USA, and Australia revealed 3 deletions and 93 mutations across the genome of SARS-CoV-2 [17]. Notably, recent research highlighted that mutations in the non-structural protein (NSP)-2 and 3 may be involved in the infectivity and differentiation of the SARS-CoV-2 from SARS [18]. Compared with SARS and MERS, SARS-CoV-2 has higher transmission and infection rate [19]. Based on the genome sequence and phylogenetic tree analysis, bat has been supposed as the natural host for the origin of the virus and might be transmitted to humans via an intermediate host [20].

Molecular modeling studies have revealed similarities between the spike protein (receptor-binding domain) of SARS-CoV1 and SARS-CoV2, a protein through which the virus binds to the cell receptor known as angiotensin-converting enzyme-2 (ACE-2) for getting entry [21–23]. However, genetic and structural analysis has also revealed a strikingly different spike protein of SARS-CoV-2, suggesting that the protein has a specific furin-like cleavage site [24], which is expressed in many human tissues including lungs, liver, and small intestine. This feature enables the virus to potentially infect multiple organs [25].

## Immune dysregulation in COVID-19 patients

### Changes of immune cells

**Figure 2.**
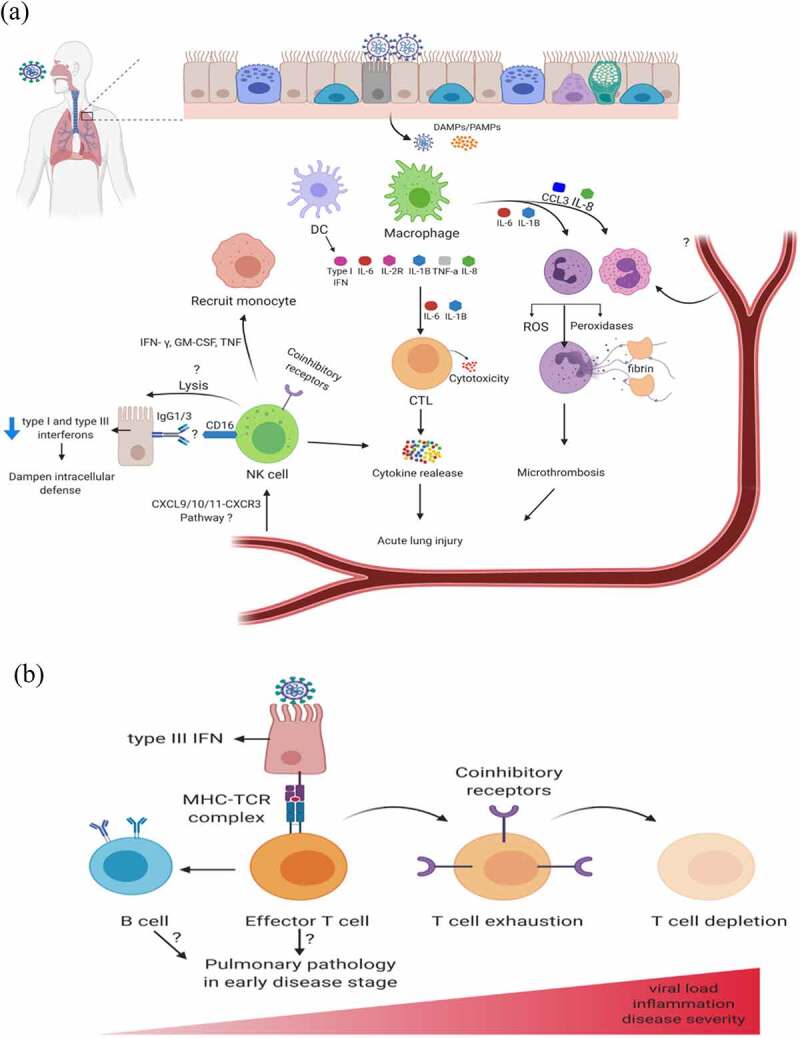
**Pathogenic mechanism of SARS-CoV-2 and activation and alteration in immune cells post-infection**. (a) DAMPs and PAMPs derived from virus results in the activation of macrophage. Followed by the downstream production of cytokines. IL-6 and IL-1b results in the recruitment of CD8 + T cells and neutrophils. Activated T-cell directs the release of cytokines and cellular cytotoxicity, which can contribute to tissue inflammation. Cytotoxic T cells result in the production of cytokines, which may cause lung injury. CCL3 and IL-8 can induce eosinophil and neutrophil recruitment leading to NETosis and microthrombosis, which may result in lung injury. NK cells might infiltrate from the blood via the CXCL9/10/11-CXCR3 pathway, CXCL9/10/11 is derived from monocyte. Inhibitory receptors such as NKG2A is also increased on NK cells and CD8 T cells, which inhibits cytotoxicity. Besides, elevated expression LAG3 and on NK cells in COVID-19 patients may also contribute to viral escape. In addition, high expression of IL-6 may also reduce NK cell numbers. Dendritic cell secretes Type 1 IFN and is supposed to play a role in control of viral infection. (b) A nonresponsive cell state is driven by the persistent antigenic T cells activation by the epithelial cells leading to T cell exhaustion, which is accompanied by lymphodepletion in severe patients. T cells overexpress markers of exhaustion including PD-1 and Tim-3. B cells-induced pulmonary pathology during SARS-CoV-2 infection may also contribute to lung injury

Coronavirus infection, in particular, SARS-CoV-2 might activate both innate and adaptive immune responses in patients. However, the knowledge of the dysregulated immune response to COVID-19 is not well explained. Lymphopenia is a common feature of SARS-CoV-2 infection in severe COVID-19 patients, including a drastic reduction in T-cells (CD4^+^ and CD8^+^) and CD19^+^B cells (Figure 2b) [5,8,26,27]. Emerging evidences have shown that severe patients had a low population of CD4^+^ and CD8^+^ T cells, while a reduction in T and B cells in non-severe patients has also been reported [28]. Intriguingly, a negative correlation between the viral RNA and CD4^+^ T cells and CD8^+^ T cells was observed in severe patients [29], suggesting that the reduction in lymphocytes population influenced by the SARS-CoV-2 viral RNA load was closely associated with disease progression [30]. Monitoring the lymphocyte count by means of detecting the viral load from a nasopharyngeal swab or any other specimen might reveal about COVID-19 prognosis [30]. Studies have demonstrated that severe cases had a lower proportion of CD45RA^+^ naïve Tregs (nTregs) and higher proportion of CD45RO^+^ memory Tregs (mTregs) compared with moderate cases [31]. Moreover, the percentage of naïve helper T cells (CD3^+^CD4^+^ CD45RA^+^) increased in severe patients, whereas the population of memory helper cells (CD3^+^CD4^+^CD45RO^+^) decreased [8]. The percentage of CD28^+^ cytotoxic suppressor T cells (CD3^+^CD8^+^CD28^+^) was significantly decreased in severe cases [27].

Another immune dysregulated feature is the remarkable increase in neutrophil-to-lymphocyte ratio (NLR) [8,32], which usually depicts a higher disease severity and poor clinical outcome [33]. NLR may also work as an independent prognostic marker to predict COVID-19 disease severity [34]. In a retrospective study of the 55 COVID-19 patients, transcriptional profiling of the immune cells revealed excessive neutrophils count and the associated NET. Excessive neutrophil count and NET coincided with lesion progression in the lungs [35]. In addition, neutrophilia was reported in several large studies reported [36]. Several studies have reported the decrease in CD16^+^/CD56^+^NK cells in peripheral blood of COVID-19 compared with healthy individuals [37]. However, NK cells seem not to be involved in the disease progression from mild to severe type of COVID-19 patients, as not obvious difference was observed between the two cohorts [38]. It has been demonstrated that IFN-γ-producing CD4^+^T cells tended to be lower in severe cases than moderate cases and correlated with disease severity of COVID-19. However, IFN-γ-producing CD8^+^ T cells and IFN-γ-producing NK cells were comparable between severe and moderate cohorts of COVID-19 [37]. Th17 cells might also play a redundant role in the pathophysiology of COVID-19 [39–41]. However, there was a lack of clinical data with direct determination of the proportions of Th2 and Th17 in the peripheral blood of COVID-19 patients up to date. In this regard, the differentiation status of Teff cells after the first or second infection of SARS-CoV-2 needs further investigations. In addition, T cells from patients with disease progression exhibited higher expression of the exhausted marker PD-1 and Tim-3 [42]. Restoration of the immune cell number and normalization of the exhaustion marker expression, such as NKG2A on T cells and NK cells have been reported in fully recovered or recovering patients [43,44]. Besides, non-severe patients who are persistently positive for SARS-CoV-2 has an improved immune cell profile compared to newly diagnosed and upon the patient recovery, the number of lymphocytes subpopulations increased to normal [45].

### Dendritic cells (DCs)

DCs play a crucial role in the interaction of both arms of immunity and the substantial loss of function of DCs could lead to the delayed T cell response in patients with COVID-19. Owing to the ACE2 surface expression particularly by the dendritic cells residing in the lungs, these cells can be directly infected by SARS-CoV-2 [46,47]. A novel infection route through CD147-S protein interaction has been recently described [48]. DCs express CD147 [49] further verifying the ability of direct target by the SARS-CoV-2. The transcriptome analysis of whole lung tissue demonstrated the higher expression of dendritic cell-specific intracellular adhesion molecule-grabbing integrin (DC-SIGN) in elderly patients aging above 60 years [50]. These findings describe that increased expression of cell receptor or other associating factors may aid in the pathogenesis of severe coronaviruses infection. Plasmacytoid dendritic cell (pDC) is the main type I (IFN-I)-producing cells providing immunity in viral infections. Type I IFN produced by these cells both facilitate the removal of the virus by macrophages and promote pDCs for priming immune response [51]. A case-study of 47 years old mild patient with full recovery within one-week post symptoms manifestation highlighted the reduction in blood CD16^+^CD14^+^ monocytes count on days 7, 8, and 9 days when compared with cells count from healthy individuals, suggesting the possibility of the migration and distribution of these cells at infection site [52]. These cells originated from human peripheral blood monocytes shows the physiological characteristic of DCs [53,54]. These evidences suggest the involvement of DCs in protection against SARS-CoV-2 in recovered patients with COVID-19 [52]. Like the other coronaviruses, e.g. human coronavirus 229E (HCoV-229E) selectively destroying DCs [55], SARS-CoV-2 would have developed immune evasion strategies to rescue against the killing by the host DCs. Another possible immune evasion mechanism is the selective infection of immature monocyte-derived DCs. The maturation state is functionally important in stimulating T cells [56]. Therefore, thearresting the cells in its immature state due to infection [57] may lead to a delay in the activation of T cells, promoting the dissemination of the virus. Based on the lesson learnt from other coronaviruses’ infection, the role of DCs in COVID-19 immunopathogenesis can be hypothesized. In COVID-19, an increased loss of pDC along with a reduction in NK level could result in a robust annihilation of the innate immunity [58,59]. Corroborating the role of DCs in immunopathology, a latest study highlights the negative effect of SARS-CoV-2 infection on DCs count and function characterized by a 10–20% reduction in monocyte-derived DCs viability 72 hours after virus inoculation [60]. Regarding the modulation of the DCs function by SARS-CoV-2, studies documented that both monocyte-derived DCs from a healthy individual with COVID-19 and CD11_C_^+^ conventional dendritic cells (CDCs) derived from acute COVID-19 patients stimulated with proinflammatory cytokines failed to efficiently produce IFN-α and IFN-β [59,60]. The dysregulated functionality of DCs in COVID-19 is associated with viral antagonism of the phosphorylation of signal transducer and activator of transcription 1 (STAT1), which is a key regulator of type I, II, and type III IFN signaling pathway [60]. In lines of these findings, inhibition of DCs number and function (IFN signaling) by SARS-CoV-2, which diminish the progression from innate to adaptive immunity, may represent an immune evasion mechanism of SARS-CoV-2 [61]. Based on all these evidences, it is presumable that treatment strategies focusing on augmentation of the DCs function would facilitate both viral removal and better prognosis.

### Monocyte/macrophage

Monocytes account for about 5–9% of the total peripheral leukocytes counts, stay in the system circulation for 1–2 days followed by the differentiation into tissue-resident macrophages [62]. Macrophages are abundantly present in the body performing various vital functions including immune surveillance and tissue homeostasis [63]. Nevertheless, the aberrant hyperactivation of macrophages may cause immunopathogenesis [62,64]. In the case of other coronavirus infections, the direct disruption of lung epithelial cells and macrophages or indirectly the activation of inflammatory mediators resulted in disease severity [65]. SARS-CoV-2 may likely use a similar route of infection as utilized by the other coronaviruses. Surprisingly, the monocytes obtained from the COVID-19 positive patients are found to be actively expressing ACE2 receptor, suggesting the possibility of direct infection of the monocytes thereby leading to abrogated viral replication and a delayed type I IFN signaling [66]. Hence, SARS-CoV-2 may directly infect monocytes and macrophages in patient with COVID-19 [67].

The excessive activation of monocytes/macrophages and CRS is associated with disease gravity and the related complexities COVID-19 [68]. The exaggerated expression of chemokines has been evident in lung tissue samples isolated from COVID-19 patients, which could contribute to pulmonary dysfunction by mediating the trafficking of the leukocytes to the lungs. Leukocytes alveolar infiltrates in COVID-19 patients were mostly consisted of macrophages and monocytes [69]. Therefore, alveolar macrophages may recruit immune cells and may promote the local inflammation in severe COVID-19 patients. Elevated proinflammatory cytokines and chemokines in COVID-19 patients from monocytes and macrophages can escalate the pathogenesis. Although the difference in peripheral blood monocyte/macrophage between COVID-19 patients and healthy donor is not remarkable, there is a great degree of the activation of monocyte/macrophages activation. The macrophages undergo functional polarization in response to the tissue microenvironment and can adopt either the classically activated, pro-inflammatory (M1 macrophage) or alternatively activated, anti-inflammatory (M2 macrophages). M1 macrophages secrete proinflammatory mediators including IL-6, IL-1β, and TNF‐α to regulate the local tissue and immune responses. Increased IL-6 in the serum, a main feature of SARS-CoV-2 infection is associated with ARDS [70]. These findings were consistent with the autopsy findings of three COVID-19 patients that showed the accumulation of M1 macrophages in the lungs [69]. The high percentage of CD14^+^CD16^+^ monocytes in severe COVID-19 patients have been demonstrated in two studies [71,72], which are specialized in producing inflammatory mediators including GM-CSF and IL-6, IL-10, and TNF-α.

Moreover, according to the model presented by Zhou et al., upon infection, CD4^+^ T cells are robustly activated to become Th1 cells and produce a response, which induces CD14^+^CD16^+^ monocytes. These abnormal Th1 cells and inflammatory monocytes may move to the lung tissue and induce an immune-damaging role subsequently leading to lung injury and rapid mortality [71]. These findings are further supported by the characterization of the lung immune microenvironment through single-cell RNA sequencing, which demonstrates the predominance of the FABP4^+^ alveolar macrophages in the severely injured lungs. FABP4^+^ alveolar macrophages produce a higher level of interferon-stimulated genes and chemokines and thus leads to hyperinflammation [32]. These results suggest the association of high level of GM-CSF and IL-6 released by monocytes with cytokine storm [67].

Despite the lack of difference in monocyte count between the normal and COVID-19 patients, morphological and physiological changes were observed in COVD-19 patients particularly those requiring intensive care. These monocytes larger than normal were characterized by mix M1/M2 polarization, relatively elevated CD80^+^ and CD206^+^ expression, and higher secretion of IL-6, IL-10, and TNF-α [67]. Extreme myeloid cell compartment changes during COVID-19 were also reported by a multipronged approach characterized by an elevation in HLA-DR^hi^CD11c^hi^/HLA-DR^hi^CD83^hi^ inflammatory monocytes with a strong antiviral IFN-signature in mild COVID-19 patients. Contrarily, the severe patients were marked with HLA-DR^lo^ dysfunctional monocytes with clear indication of emergency myelopoiesis and dysfunctional mature neutrophils [73]. Besides altered morphology of monocytes, SARS-CoV-2 infection may also be associated with macrophage and lymphocytes pyroptosis [74]. This is in line with the previous report demonstrating the activation of NLRP3 inflammasome pathway by viroporin-3a and secretion of IL-1β by the macrophage upon SARS-CoV infection [75]. Collectively, SARS-CoV-2 infection result in altered monocyte/macrophage count, morphology, and physiology, which may result in severe COVID-19.

### Complement system

The complement system (CS) plays a substantial role in the host immunity to COVID-19. The robust activation of the CS has also been detected in COVID-19 patients, which might contribute to the pathogenesis of ARDS [76,77]. Patients with moderate and severe COVID-19 have increased level of C5a and sC5b-9 level in plasma [78]. Notably, the elevated level of sC5b-9 or membrane attacking complex (MAC) is also evident in patients with normal C5a owing to its robust clearance, thus sC5b-9 may work as a predictor of CS activation.

To circumvent the detection by the CS, virus employ encoded proteins, suggesting the antiviral role of the CS. Nevertheless, C3a and C5a with proinflammatory properties may initiate inflammatory cells recruitment and induce neutrophils [79]. Gao et al. reported that N protein mediates the attachment of SARS-CoV-2 to mannose-associated serine protease (MASP-2), which is a serine protease involved in the activation of the lectin pathway of the CS. This complex in turn activates MAC and ultimately leads to severe inflammatory lung injury [77]. Similarly, the accumulation of MBL, MASP-2, C3, C4a, C4d, and C5b-9 is evident in the vascular endothelium of COVID-19 with lung injury [80]. Moreover, the increased level of von Willebrand factor release from the endothelial cells in severe COVID-19 patients contribute to the damage of vascular endothelium [81,82].

As a therapeutic approach to treat acute lung injury, the inhibition of C3a and C5a has been found very effective and particularly anti-C5a antibody was documented to improve the adverse clinical symptoms associated with MERS-CoV [76,79]. COVID-19-induced acute lung injury can be ameliorated by inhibiting C5a with anti-C5a monoclonal antibody [77]. Increase NETosis and tissue factor activity have been observed in severe COVID-19 patients. Inhibition of thrombogenesis or NETosis or blocking C5aR diminishes CS hyperactivation-induced acute lung injury [77]. In addition, the higher level of C3 and C4 in the cerebrospinal fluid of men of age 20–50 may explain the higher mortality rate in men than the women due to hyperinflammatory and tissue-damaging function of CS during SARS-CoV-2 infection [83]. Interestingly, the C3 knockout mice infected with SARS-CoV prevented acute lung injury formation [79]. Furthermore, the upregulation of CS genes C1S and C1R and the downregulation of the genes regulating CS (C1q) during COVID-19 further corroborate the role of CS hyperactivation in immunopathology.

### Eosinophils and basophils

Like the other infections, a reduced percentage of eosinophils and basophils is evident in COVID-19 [84]. Remarkably, in a retrospective study of 85 fatal cases of COVID-19, 81.2% of the patients at the time of admission had lower absolute eosinophil counts (<0.02 × 10^9^ cells/L) [85]. Similarly, in a cohort of COVID-19 patients, eosinopenia was evident in more than 78.8% of patients [86]. Based on decreased eosinophil count in 52.9% COVID-19 patients of the total patients tested, Zhang et al. suggested that eosinopenia along with lymphopenia could be as an indicator for diagnosis [87]. The eosinophil level in systemic circulation decreased dramatically in 71.7% COVID-19 patients compared to other types of pneumonia patients with a diagnostic value higher than NLR [88]. Notably, in a small cohort, the eosinophil count was low at the time of initial hospitalization who were treated with lopinavir, but the level increase constantly and returned to the normal prior to the discharge, which suggest that increase eosinophil count may serve as a predictor of COVID-19 improvement [32]. The role of eosinopenia in the pathophysiology of COVID-19 is obscure and is likely dependent on multiple factors, including the prevention of egress of eosinophil from the bone marrow, decreased chemokine receptors expression [89,90], and/or the apoptosis of eosinophil. During acute infection, the apoptosis of eosinophil is induced by type I IFN [91]. Notably, it is not determined that whether the acquired eosinophilia in COVID-19 underwrites for the disease progression or may serve as a predictor of disease severity, however it is obvious that pulmonary eosinophilia is not a part of the pulmonary pathological findings specified to SARS-CoV-2 infection [92].

### The kinetics of hypercytokinemia

**Figure 3.**
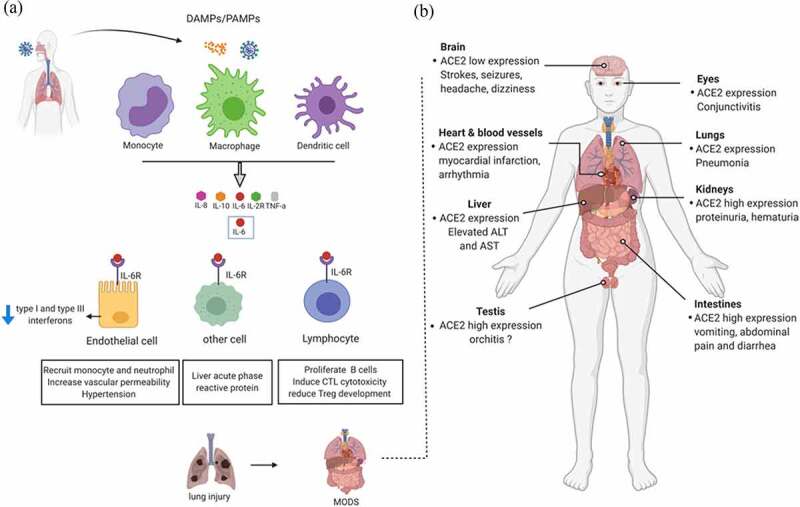
**Illustration of the SARS-CoV-2 pathogenesis**. (a) Virus binds to ACE2 receptor and enters into the cell through membrane fusion or endocytosis and release its RNA genome. Viral infection results in the activation of monocytes, macrophages, and dendritic cell instigation to release several cytokines and among these cytokines, a key player IL-6 can activate signaling pathways. These pathways are associated with lymphatic cell changes characterized by the proliferation of B cells, induction of CTL cytotoxicity, and reduction of Treg development. In addition, increase recruitment of monocytes and neutrophils, increase vascular permeability, and hypertension are also caused by the Trans signaling pathway activated by IL-6. The subsequent elevated cytokine production aid to pathophysiology in COVID-19. (b) ACE2 expression in organs and systems contribute to COVID-19. ACE2 is highly expressed on the GI tract, kidneys, and testis. Notably, expression of ACE2 varies remarkably from cell to cell in the same organs, for example, ACE2 is poorly expressed on bronchial epithelial cells compared to the higher expression on alveolar epithelial cells. Hepatocytes, Kupffer cells endothelial cells lack ACE2 expression but cholangiocytes expressed at a detectable level

Cytokine release syndrome (CRS) is a key feature of severe COVID-19 [93] and increased serum level of IL-6 correlates with ARDS and adverse clinical outcomes. CRS is usually initiated by macrophages, dendritic cell, NK cell, and T cell, in response to pathogen-associated molecular patterns. The mechanism of CRS resulting in ARDS, is illustrated (Figure 3a). COVID-19 patients have higher serum levels of IL-6, IL-1β, soluble IL-2 R, IL-8, IL-10, IL-17, and TNF-α [8]. Huang et al. reported that IL-2, IL-7, IL-10, granulocyte-colony stimulating factor (G-CSF), interferon-inducible protein-10 (IP-10), monocyte chemoattractant protein-1 (MCP-1), macrophage inflammatory protein (MIP-1A) and TNF-α were increased in severe patients [5]. Elevated levels of IL-6, IL-10, and TNF-α were associated with disease severity [5]. Additionally, COVID-19 patients have increased acute-phase proteins in serums, including procalcitonin (PCT), serum amyloid A (SAA), C-reactive protein (CRP), and serum ferritin (SF) [5,8]. Moreover, SAA has been reported as a novel indicator to evaluate the severity and prognosis of the disease [94,95].

The abnormal activation of host leukocyte most commonly neutrophils exacerbates the host immune response, which predicts poor prognosis in COVID-19 patients [31]. Moreover, NLR is an independent risk factor for severe disease [96]. Furthermore, the neutrophil tissue infiltration and the associated tissue damages are evident in two studies involving autopsies [97,98]. The elevated level of neutrophils and NLR could be the potential source of neutrophil extracellular trap (NET). NETs are thread-like structures formed from DNA and protein, which are expelled from the neutrophil to capture the pathogen. These inflammatory cytokines regulate the activity of neutrophil and it is worthy to mention that NET induces macrophage to release IL1B, which in turn facilitate NETosis in various diseases (Figure 2a) [99]. These evidences suggest that under abnormal immune homeostasis, a coordination loop develops between macrophage and neutrophil, which can lead to redundant progressive inflammation. If such a correlation is formed in severe COVID-19 patients, the elevated production of NETs and IL1B could lead to a worse immune outcomes. Therefore, potential targeting of IL6 [100] induced by IL1B is an exciting therapeutic approach in COVID-19 [101].

Upon SARS-CoV-2 infection, monocytes, macrophages, and dendritic cells are activated [102]. IFN-γ might be involved in the initiation of cytokine storm in CRS in COVID-19 patients [103]. Cytokines including IL-6, IL-10, TNF-α, and IL-2 R produced by macrophages trigger and amplify the vicious cytokine storm. The dramatic release of these cytokines make the differentiation and function of lymphocytes and the function of endothelial of the blood vessels abnormal, via signaling transduction [93]. NK cells might also play an essential role in non-redundant role in this process [104]. Numerous studies have reported a reduced number of NK cells in COVID-19 patients, which is associated with disease severity [105,106]. Most of the NK cells present in the lungs are nonresident [107,108], which are infiltrated and it is believed that the CXCR3 pathway mediates NK infiltration as a result of influenza infection [109]. Besides, SARS-CoV-2 infected human lung tissue showed high CXCR3 ligand in vitro [110], and also lungs of COVID-19 patients showed increased monocyte producing CXCR3 ligand [32]. These findings suggest that CXCR3 pathway might promote the recruitment of NK to the lung (Figure 2a). The cytotoxicity of NK cells is regulated by their expression of inhibitory and activating receptors. The mechanism of NK cell activation either by mean of the expression of NK receptor ligand expression, such as NKp46 on the SARS-CoV-2 infected cells is a subject of investigation. In addition, during SARS-CoV-2 infection, IgG1 and IgG3 antibodies are secreted [111], which may cause the induction of CD56dim CD16^+^ NK cell activation by recognition of the antibodies through Fc receptors (Figure 2a). Such interaction may activate not only cytokine release but also infected cell lysis by NK cells in a process called antibody-mediated cellular cytotoxicity (ADCC), which is evident in influenza infection [112].

Activated NK and T cells further promote the recruitment and activation of monocyte-derived macrophages through the production of Granulocyte-macrophage colony-stimulating factor (GM-CSF), TNF, and IFN-γ (Figure 2) [113]. Moreover, upon infection of Chinese hamster ovary cells by the SARS-CoV-1 results in the production of anti-S IgG that could cross-react with SARS-CoV-2 S glycoprotein with the capability of NK-mediated ADCC response [114]. It might be speculated based on these findings that NK cell activation is associated with clearing viral infection but also aid in cytokine storm in ARDS. Finally, rapid damage of lung tissue and other organs occurs, leading to ARDS, sepsis, as well as multiple organ failure [115].

### Relationship between viral load, host inflammation, and disease severity:

T cell contributes to COVID-19 hyperinflammation. T cells are the frontline defender to control viral infections and a dysregulated T cell immune response may lead to immunopathology and ultimately result in severe disease outcome. Likely, increase in the frequency of PBMC GM-CSF^+^ CD4^+^ T cells produces a high level of proinflammatory cytokines including IL6 and IFN-γ is evident in severe disease [116]. Notably, PBMC GM-CSF^+^CD4^+^ T cells have also been implicated in autoimmune disorders [117]. Another feature of COVID-19 altered distribution regulatory T cells (Tregs) subsets characterized by lower proportion of nTregs and higher proportion of mTregs in severe cases [8]. Treg is believed to play a role in the reduction of ARDS in mouse models [118]. These findings suggest that therapies that reestablish Treg homeostasis may be beneficial. Besides, mounting studies demonstrate that elevated number of activate T cells (CD4 and CD8) showing the tendency toward the exhausted phenotype, is insistent in COVID-19. These cells overexpress the inhibitory markers suggesting a reduced polyfunctionality and cytotoxicity to clear the virus-infected cells over time [28].

Taken together all these evidences suggest that a dysfunctional immune response characterized by reduced lymphocytes count, hyperinflammation may results in increase viral load. The abundance of SARS-CoV-2 in plasma is predictive of worse respiratory disease severity and mortality [119,120]. Intriguingly, the abundant plasma viremia has association with reduced absolute lymphocyte number, higher inflammatory markers expression [119].

## System pathology of COVID-19

Mounting evidences suggest that in SARS-CoV-2 infection, apart from the lungs, other tissues and organs can be also damaged (Figure 3b). Majority of the old-aged patients tend to have the underlying comorbidities, such as cardiovascular disease, hepatic disorders, kidney disease, and malignancies [5,121,122], which increases the severity of COVID-19.

### Respiratory system

Lung is the primary target for the corona viruses. Chen et al. 2020 found 75% of the patients with bilateral pneumonia, whereas only 25% with unilateral pneumonia in a cohort of 99 COVID-19 patients [122]. Prominent desquamation of pneumocytes, formation of hyaline membrane and pulmonary edema and mononuclear inflammatory infiltrates were observed in the lungs, which indicates the occurrence of ARDS [26]. Hyaline membrane formation, diffused type II pneumocytes hyperplasia, epithelial damage, and fibrin exudates were observed in COVID-19 patients postmortem biopsies [123]. The same group performed the lobectomies of the lung for two pre-symptomatic COVID-19 patients with underlying lung adenocarcinoma and found COVID-19-associated pathological changes in the lungs characterized by edema, proteinaceous exudate, pneumocytes hyperplasia, multinucleated syncytial giant cells, but without the formation of hyaline membrane [115].

### Cardiovascular system

Cardiac complications including heart failure, myocardial infarction, arrhythmia has been observed in COVID-19 patients. The elevated level of Cardiac Troponin I (Trop I), a marker of myocardial injury is evident in patients [121,124,125]. A case report of a single COVID-19 patient with no previous underlying cardiovascular disease reported the features of fulminant myocarditis [125]. Similarly, coronavirus infection can cause myocarditis and even congestive heart failure [126]. The autopsy examination of a COVID-19 patient shows a few interstitial mononuclear inflammatory infiltrates without a substantial histological changes in cardiac tissue, suggesting that SARS-CoV-2-associated infection may unlikely impair the heart [26]. The robust recovery of cardiac physiology and no change in the viral load may speculate the role of cytokine storm (IL-6 elevation), which increases the vascular permeability leading to edema and eventually the thickening of the septum. Another potential possibility is the expression of ACE2 receptor on the vascular endothelial cells, which may allow the entry of SARS-COV-2 [127,128]. Notably, a few interstitial mononuclear inflammatory infiltrates were observed in heart tissue but without any obvious damage to the heart tissue [26]. Pathophysiological changes in the heart may be the result of direct virus replication in myocardium or an indirect systemic response from respiratory problems or virus-induced exaggerated immune response.

### Gastrointestinal system (GIS)

Biochemical-based detection of liver dysfunction characterized by significantly elevated serum aminotransferases and bilirubin is evident in a large cohort of COVID-19 patients [129]. Based on findings from case studies in China, 14–53% of COVID-19 cases had an aberrant levels of alanine aminotransferase (ALT) and aspartate aminotransferase (AST) with the disease progression [130]. Elevated serum AST was evident in a cohort of severe COVID-19 patients compared with mild-patients [5]. Another study reported mild histological liver abnormalities characterized by mild sinusoidal dilation, lobular lymphocytic infiltration, and focal macrovesicular steatosis. Nevertheless, limited hepatic histological changes were observed and only one patient’s liver tissue was tested positive for viral infection [123]. The examination of liver biopsy samples from only a 50-year-old man COVID-19 patient showed microvesicular steatosis and mild lobular and portal activity [123]. Different degree of liver dysfunction characterized by a mild-to-moderate elevation in ALT or AST was observed in 43 patients in a cohort of 99 patients investigated [122]. Moreover, confirmed COVID-19 patients in the subclinical phase appeared to have a low rate of AST abnormality compared to the patients after the onset of the symptoms, however, with a later presentation, a greater liver dysfunction is not evident [131].

It is not yet concluded whether the liver derangement is the result of direct SARS-CoV-2 infection or the effect of hepatotoxic drug or systemic inflammatory response. It is important to note that elevation in liver function biomarker is associated with respiratory viral infections and it is believed that hepatic damage is caused by the cytotoxic T cells and Kupffer cells [132]. The drug-induced hepatoxicity as a factor contributing to liver dysfunction in COVID-19 is important to consider but the mild abnormal liver test is present at baseline in many COVID-19 patients before the start of medication [133]. Evaluation of the cell type-specific expression of ACE2 via RNA-sequencing in two independent cohorts revealed the high expression of ACE2 in bile duct epithelium but not necessarily in the hepatic cells [134]. This finding suggests that SARS-CoV-2 associated liver damage might not be the direct targeting of the hepatic cells by the virus, but instead by cholangiocyte dysregulation and dysfunction, which is crucial in liver regeneration and immune response [135]. In addition, it is more likely that virus-induced cytotoxic T cells and a dysregulated innate immunity may contribute to the collateral liver damage in COVID-19 patients. The detection of viral RNA in rectal swabs [136,137] and stool sample of COVID-19 patients [7,138,139] raises the concern of the GIS infection and its fecal to oral transmission [140]. Moreover, the high expression of ACE2 in GI epithelial cells speculate its infection by SARS-CoV-2 [141,142]. The staining and detection of nucleocapsid protein in stomach and intestinal epithelial cells demonstrate the ability of the viral to infect glandular epithelial cells [143]. The most common GIS symptoms include nausea and/or vomiting, abdominal pain, and diarrhea. It is rather of concern that several patients (23.3%) in a cohort tested persistently positive for viral RNA in their fecal materials after respiratory clearance as evidenced by the negative result from nasal swab samples [144]. Similarly, in an evaluation of 10 pediatric patients, 8 children remained positive and negative on rectal swab and nasal swab, respectively [137]. The persistence of the virus in the stool sample may reflect the abundant viral shedding from the GI tract, which may cause the long-lasting of the virus even when the clinical symptoms are resolved [138]. Nevertheless, the detection of viral RNA in other systems outside the respiratory system does not necessarily signify the persistence of viral infection since the clinical importance of the extrapulmonary detection of viral RNA is mysterious in the present time.

### Central nervous system (CNS)

SARS-CoV-2 infected patients also show neurological symptoms such as headache, dizziness, nausea and vomiting, and loss of taste and smell, which speculate that the novel coronavirus can invade the CNS. Metagenome sequencing of the collected cerebrospinal fluid sample from a patient confirmed the presence of SARS-CoV-2 causing viral encephalitis (Hospital BD; http://www.bjdth.com/html/1/305/307/index.html?messageId=3665 (accessed Mar 04)). The analysis of a cohort of COVID-19 patients showed that 78 patients out of 214 (36.4%) had neurological manifestations. Severe COVID-19 patients had more neurological problems characterized by acute cerebrovascular disease, retarded consciousness, and muscle lesions [145]. More recently, SARS-CoV-2 was detected in the cerebrospinal fluid of a patient with COVID-19- associated meningitis [146]. These findings highlight the potential of SARS-CoV-2 to cause NS damages [146]. The neuroinvasive mechanism of SARS-CoV-2 is not well studied. Owing to the expression of ACE2 on the brain cells and endothelial cells [147], ACE2 receptor-based entry of the viral into the brain cells [148,149] epitomize the key, but not exclusively. Under normal conditions, the expression of ACE2 in CNS is very low [150], but this might not completely rule out the inability of viral entry into the brain cells via receptor-mediated endocytosis. Therefore, non-ACE2 pathways for neural cell infection cannot be excluded. The propensity of β-coronaviruses invading the CNS and inducing neurological manifestations in experimental animals is documented for SARS-CoV [151] and MERS-CoV [152]. Mounting evidences suggest that human coronaviruses initially infect peripheral nerve terminals followed by trans-synaptic transfer to the CNS [153–155]. Mechanistically, whether SARS-CoV [156] or MERS-CoV [152] could invade the brain through olfactory nerves when administered intranasally in mice. In addition, diagnosis through electron microscopy, immunohistochemistry, and real-time PCR of the patients has confirmed the presence of SARS-CoV [156]. The disseminating virus once enters into the systemic circulation can invade the cerebrum as evident in SARS-CoV infection [156]. The slow neurotropism of the virus may facilitate its interaction with ACE2 expressed on capillary endothelium and subsequently initiate the viral shredding cycle from the capillary endothelium, which may damage the endothelial lining. Rupturing of endothelial capillaries is followed by bleeding in the cerebral tissue, which can lead to deleterious results in patients. Another possibility is the viral dissemination in cardiorespiratory centers causing its derangement [155,157]. The Last but important aspect is the massive release of cytokines and chemokines, which can compromise the integrity of the blood-brain barrier and promotes neuroinflammation, which may result in a disturbed brain homeostasis and neuronal death [158].

### Ophthalmological system

The expression of ACE2 on the corneal epithelial and endothelial cells, and conjunctival epithelium may act as a gate for the entry of coronavirus in eye [159], which can potentially cause the infection of the conjunctival epithelium [160]. Infection of the ocular surface in COVID-19 patients is reported by several studies. COVID-19 patients with conjunctivitis may have SARS-CoV-2 in tears and conjunctival secretions [161]. Zhou et al. performed real-time RT-PCR on the conjunctival swab samples from 4 patients with novel corona virus pneumonia and found one patient positive and three patients probable positive for the COVID-19. The four patients had no previous history of ocular syndrome [162]. A Chinese health expert infected with COVID-19 reported on a social medium that the route of transmission could be conjunctiva [163].

### Urinary and reproductive systems

Two studies posted on medRxiv documented a remarkable percentage of patients with signs of kidney dysfunction characterized by proteinuria, hematuria, and elevated level of blood urea nitrogen and serum creatinine [164,165]. Notably, patients with kidney injury had a high mortality risk compared to those with no kidney injury. The etiology of kidney injury in COVID-19 is also a result of multiple factors, which might be either caused by the direct damage of the virus to the kidney cells since the detection of the SARS-CoV-2 in patients’ urine samples [5]. Another possibility is the direction infection of the renal tubular cells [166] and kidney cells [165], which are actively expressing ACE2 receptor 100X higher than lung cells. Furthermore, kidney tissue damage may also be associated with the accumulation of virus-induced immune effectors (T cells and antibodies) or virus-induced CRS may lead to hypoxia, shock, and rhabdomyolysis to damage the renal tissue. Previous studies have reported SARS-CoV-induced testicular damage in SARS patients. SARS-CoV could cause orchitis and influence spermatogenesis. Notably, deranged immune response characterized by elevated CD3^+^T lymphocytes and CD68+ macrophages in interstitial tissue could cause orchitis [167]. The information regarding the involvement of reproductive organs dysfunction in COVID-19 is inadequate. The expression analysis of ACE2 on Leydig cells and seminiferous tubules [166] make the testicular system vulnerable to be infected by the virus SARS-CoV-2.

Sequential Organ Failure Assessment (SOFA) score is an indicator of sepsis and septic shock and imitates the degree of multiple organ failure [168,169]. Higher SOFA score (5・65, 2・61–12・23) along with other risk factors such as old age, elevated D-dimer (>1 µgᐧml^−1^) were associated with a higher number of deaths [124]. Although sepsis is usually caused by bacterial-associated infection, 40% of adults with Community-Acquired Pneumonia (CAP) had sepsis syndrome [170]. However, more than half of COVID-19 patients had developed sepsis without any underlying bacterial infection [124].

Therefore, it is important to consider that COVID-19 may also cause damage to the multiple organs mentioned above as well as to the organ system (blood and immune system), and patients’ death may be associated with the failure of any of the potential organ. Attention should be paid to treat and prevent multiple-organ injuries in the management of COVID-19 patients.

## Perspectives

MERS, SARS, and COVID-19 have outstandingly similar clinical and epidemiological features. The elevated level of pro-inflammatory cytokines and chemokine causes cell depletion, pulmonary inflammation, and extensive lung and multiple organ damage during coronavirus infection [171]. The low level of IFN-I may suppress Th1 response but facilitate Th2 [172,173]. Immune-mediated tissue damaged may be explained by the presence of highly cytotoxic CD8^+^T cells [26]. Lymphopenia in these virus-induced infections is still a subject of debate. It might either be caused directly by the virus or the result of redistribution of white blood cells [174,175] since the huge infiltration of the cytotoxic T cells in the lungs to clear the infection [176]. Of note, cytokines (IL-6 and IL-8) released upon infection of SARS-CoV-2 hamper the ability of T-cells to activate dendritic cells and macrophage and circumvent the adaptive immune response [174]. It is important to note that IL-6 suppresses the normal activation of T-cells [129]. Besides, the detection of high titer of IL-6 in the dead patient of H5N1 influenza outbreak in 1997 also depicted the role of IL-6 activation in causing lymphopenia [177]. In addition, the elevated level of markers of exhaustion including programmed Cell death protein (PD1) and NKG2A (CD94) on the cytotoxic cells such as CD8^+^ T cells and NK cells [44,178,179] predicts impairment of the immune system observed in COVID-19 patients since the immune system is shifted toward immunosuppressive Th2 [5].

The causes of CRS and its associated tissue damages are mysterious, which need further research. Various hypotheses put forwarded by the research communities are important for consideration. One of the hypotheses is that the viral replication after getting entry into the cell induce cell pyroptosis that might result in the production of massive amount of inflammatory cytokines [180]. Formation of NET by the neutrophil may also induce CRS [99]. The hyperactivation of NF-κB and STAT3 pathway either as a result direct viral infection or elevated Angiotensin 2 (AngII) in serum may result in CRS [181]. Another research supposed that antibodies production against the viral spike protein might act as a facilitator in the accumulation of immune cells in the lungs [182].

Innate lymphoid cells (ILCs) play a vital role in immunity against pathogens and maintaining tissue homeostasis in inflammation [183]. Recently, a study explained that ILCs depletion which was disrupted by the inflammatory cytokines in HIV-1 infected patients can result in the loss of integrity of gut epithelial cells [184]. ILC regulatory cells exhibit immunosuppressive function on innate immune cells through secreting IL-10 and TGF-β and prevent renal injury [185]. Similarly, ILC2 induces antibody-producing plasma cells and play a role in stomach tissue protection [186]. ILC2 and ILC3 express ACE2 significantly [187]. These findings suggest the potential targeting and depletion of the ILCs by the SARS-CoV-2, as well as its subsequent depletion and associated chronic inflammation.

### Therapeutic interventions

As described above that the high mortality in coronavirus-associated deaths is due to high virus titer and cytokine storm, which results in an exaggerated immune response. Based on the lesson learned from SARS and MERS treatments, targeting the viral through antiviral drugs in the early stages of infections as well as administration of the immunomodulators are the effective therapeutic strategies to increase the prognosis of coronavirus particularly SARS-CoV-2 infection [188–191]. The list of potential drugs of different classes including antiviral, immunomodulators are summarized in Table 1. A promising approach for the treatment of COVID-19 patients is to use mesenchymal stem cells (MSCs) owing to their pharmacological, self-renewal, and epithelial cell repair abilities. Clinical trials of MSC-based therapy have been started in China and two trials are recently in progress [192]. Another potential measure to alleviate COVID-19-associated immunopathology is to strengthen vascular permeability.

**Table 1.** List of drugs of different classes approved/in clinical crises or of potential against COVID-19

Drug	Class	Mechanism of action	References
Remdesivir	Antiviral	Adenosine analogue prodrug that interfere with virus after entry.	**[95]**
Lopinavir/ Ritonavir	Antiviral	Protease inhibitors Inhibiting human the protease of immunodeficiency virus (HIV-1) for protein cleavage, resulting in noninfectious, immature viral particles	[193]
Nafamostat	Anti-viral	Synthetic serine protease inhibitor that inhibits the fusion of membrane by decreasing Cathepsin B release	[194]
Ribavirin	Anti-viral	Synthetic guanosine Nucleoside that interfere with viral mRNA synthesis	[195]
Nitazoxanide	Anti-microbial	Affecting the survival, growth and proliferation of various viruses including coronaviruses	[196]
Ivermectin	Anti-viral and anti-parasitic	Inhibitor of the HIV integrin (IN) protein nuclear import	[197]
Anifrolumab	Anti-inflammatory (anti-interferon)	A human monoclonal antibody to type I interferon receptor subunit 1	[198]
Adalimumab	Anti-inflammatory	Anti-TNF-α	[199]
Anakinra	Anti-inflammatory (anti-interleukin)	Recombinant human IL-1β receptor antagonist	[200]
Tocilizumab	Anti-inflammatory (anti-interleukin)	A monoclonal antibody binding and inhibiting IL-6	[200]
Choloroquine	Anti-malarial	9-aminoquinolin, impair glycosylation of ACE2 receptor increasing the endosomal pH necessary for viral/host cell fusion, autophagy inhibitor	[196,201]
Baricitinib	Anti-inflammatory	Janus kinase (JAK) inhibitors, inhibit viral entry	[202,203]

Conclusively, to reduce COVID-19-associated fatalities, smart therapeutic strategies need to be designed. To this end, these strategies should aim at targeting the viral entry, alleviating the immunopathology by timely control of cytokine storm with anti-inflammatory drugs keeping immune homeostasis in mind and also lessening infiltration of inflammatory cells into the lungs as well as other organs. Of course, the development of effective vaccines and efficacious epidemiological measure to restrain the virus spread will also help in winning the battle against the COVID-19 pandemic.

## Supplementary Material

Supplemental Material

## Data Availability

None

## List of abbreviation

2019-: nCoV,
2019-: novel Coronavirus;
SARS-: CoV2, Severe Acute Respiratory Syndrome Coronavirus 2;
COVID-19,: Corona Virus Disease 2019;
ARDS,: Acute Respiratory Distress Syndrome;
ACE-2,: Angiotensin Converting Enzyme 2;
TNF,: Tumor Necrosis Factor;
IL,: Interleukin; IFN, Interferon;
NK,: Natural Killer;
MERS,: Middle East Respiratory Syndrome;
NLR,: Neutrophil-to-lymphocyte ratio;
SF,: Serum Ferritin;
PCT,: Procalcitonin;
CRP,: C-Reactive Protein;
SA,: Serum Amyloid;
G-CSF,: Granulocyte-Colony Stimulating Factor;
IP-10,: Interferon Inducible Protein-10;
MCP-1,: Monocyte Chemoattractant Protein-1;
MIP-1A,: Macrophage Inflammatory Protein;
CAP,: Community-Acquired Pneumonia;
PD1,: Programmed Cell death protein
