# Supplementary material for: Immune dysregulation and system pathology in COVID-19
Source: Virulence. 2021 Mar 23;12(1):918–36. doi: 10.1080/21505594.2021.1898790 (PMC7993139; doi:10.1080/21505594.2021.1898790)
Supplement: Supplemental Material [file KVIR_A_1898790_SM1506.zip › Document.rtf]

Supplementary figure: Statistics of COVID-19 worldwide.  (A) Distribution of cases in different countries. (B) Curve representing the pattern of increase in COVID-19 morbidities. (C) Representation of the COVID-19- associated mortalities over the period of time from January 22 to till date. The data is of 07 January 2021, 10:40 GMT+8, which is available on https://www.worldometers.info/coronavirus/worldwide-graphs/. 
