# Supplementary figures and images for: Immune dysregulation and system pathology in COVID-19
Source: Virulence. 2021 Mar 23;12(1):918–36. doi: 10.1080/21505594.2021.1898790 (PMC7993139; doi:10.1080/21505594.2021.1898790)

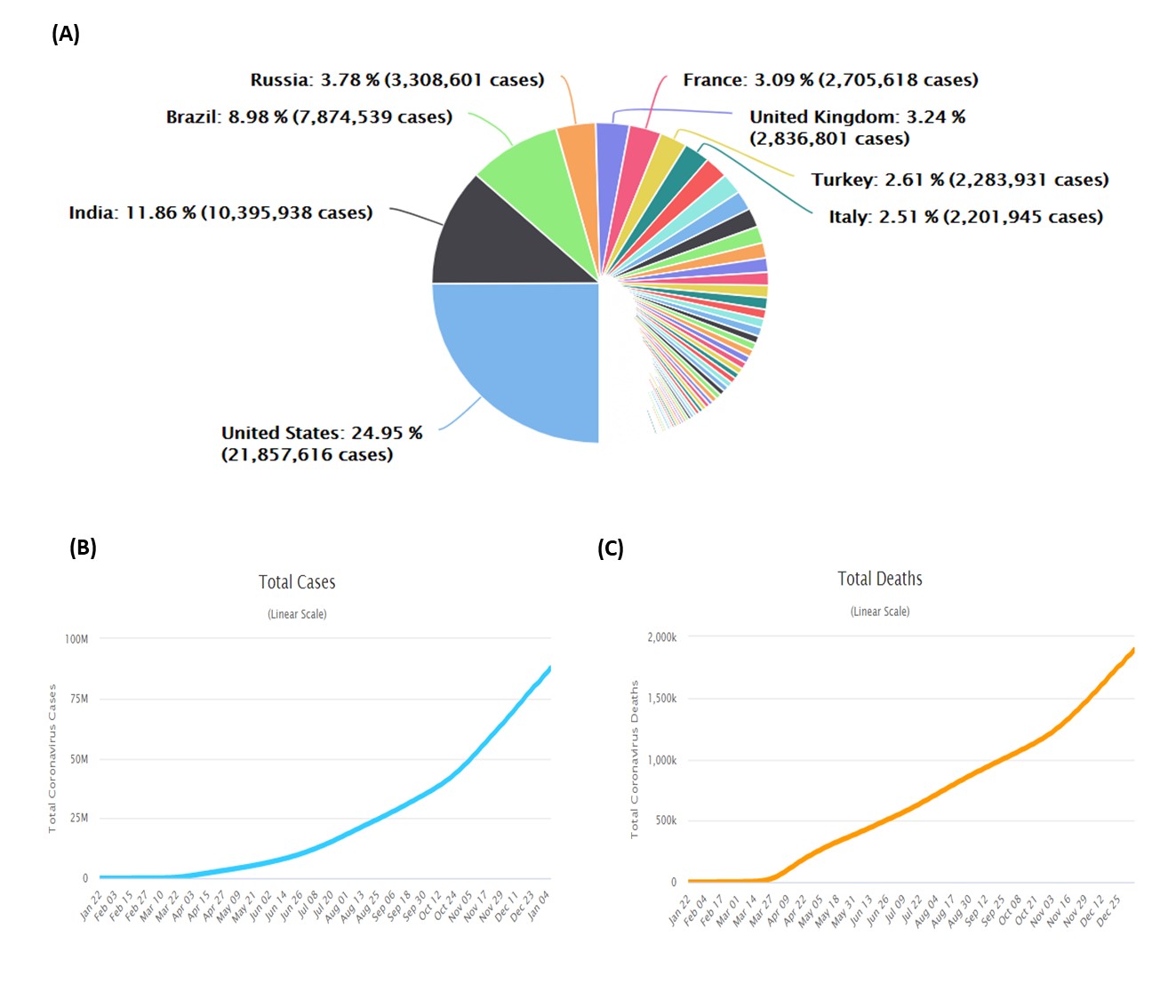

Supplement: Supplemental Material [file KVIR_A_1898790_SM1506.zip › Supplementary figure-revised.jpg]
